# Genetic subtypes of Alzheimer’s disease are related to differential biomarker and cognitive trajectories

**DOI:** 10.1002/alz.093276

**Published:** 2025-01-03

**Authors:** Jeremy A. Elman, Nicholas J. Schork, Aaditya V Rangan

**Affiliations:** ^1^ University of California, San Diego, La Jolla, CA USA; ^2^ University of California San Diego, La Jolla, CA USA; ^3^ The Translational Genomics Research Institute (TGen‐ an Affiliate of City of Hope), Phoenix, AZ USA; ^4^ New York University, New York, NY USA

## Abstract

**Background:**

Alzheimer’s disease (AD) exhibits considerable phenotypic heterogeneity, suggesting the potential existence of subtypes. AD is under substantial genetic influence, thus identifying systematic variation in genetic risk may provide insights into disease origins. We previously identified a genetic heterogeneity across two levels. The first level, which was disease‐relevant but not disease‐specific, comprised three clusters (termed “constellations”) driven by different correlation patterns in a region of extended LD surrounding the MAPT locus. The second level reflected disease‐specific genetic subtypes of AD. These structures were previously found to replicate in an independent dataset. Here, we investigate whether genetic subtypes demonstrate different patterns of biomarker and cognitive trajectories.

**Method:**

In a discovery dataset from the UK Biobank (AD cases = 2,739, controls = 5,478), we applied principal component analysis (PCA) to AD‐associated variants, allowing us to identify three constellations. Subsequently, a biclustering algorithm identified two distinct disease‐specific genetic signatures among subsets of cases not found in controls. We classified individuals with mild cognitive impairment (MCI; n = 399) from the Alzheimer’s Disease Neuroimaging Initiative (ADNI) into constellations and determined whether they were a member of either bicluster (i.e., genetic subtype) or not. Longitudinal change in cognitive performance (Preclinical Alzheimer’s Cognitive Composite; PACC), amyloid (florbetapir PET) and p‐tau (CSF) was compared between the two bicluster groups and with non‐bicluster cases.

**Result:**

The disease‐relevant constellations and disease‐specific bicluster structure was apparent in the ADNI MCI sample. Individuals with genetic signatures resembling bicluster 2 exhibited greater decline on the PACC compared to the non‐bicluster (p = 0.022) and bicluster 1 groups (p = 0.004). Bicluster 1 demonstrated a somewhat greater increase in florbetapir over time compared to the non‐bicluster group, but this difference was not significant (p = 0.129). Bicluster 2 demonstrated a significantly greater increase of CSF p‐tau over time compared to the non‐bicluster group (p = 0.041)

**Conclusion:**

These results provide additional evidence for the existence of AD genetic subtypes in an independent dataset. The genetic subtypes exhibit differential pathological accumulation and cognitive decline over time, suggesting that this genetic variation has meaningful consequences for downstream processes and disease manifestation.

